# Enhanced network load balancing technique for efficient performance in software defined network

**DOI:** 10.1371/journal.pone.0284176

**Published:** 2023-04-13

**Authors:** Evans Osei Kofi, Emmanuel Ahene

**Affiliations:** Department of Computer Science, Kwame Nkrumah University of Science and Technology, Kumasi, Ashanti Region, Ghana; Galgotias University, INDIA

## Abstract

Hash collisions and redirection of loads are major limitations for recent Hash IP algorithms. To overcome this, we propose a new Hash IP algorithm dubbed HDW in network load balancing (NLB) to increase network’s efficiency, availability and scalability. We achieve the new Hash IP load balancing algorithm via a constructive merger with weighted scheduler (WS) technique and dynamic switching of routing path (DSP). This helps to reduce delays, jitters and additionally assures some level of security owing to the hashing process. Our findings after comprehensive simulations and performance evaluation depicts that our proposed HDW algorithm is relatively efficient as against other related load balancing algorithms for software defined network.

## Introduction

Software defined network aids network operators to control the whole network constantly and holistically, irrespective of the kind of network infrastructure. Through the implementation of SDN, network programmability is improved and network nodes are distantly controlled from a centralized point. Software defined networking is assembled based on three (3) key theories which include: Programmed Networking [1], Centralization Network, Data and Control planes separation [2]. SDN works within a structured architectural build up. The architecture of SDN is divided into three (3) planes namely; Application Plane, Control Plane, and Data Plane. However, SDN faces some challenges during operation [3]. Due to the centralized nature of SDN architecture, there is traffic congestions affecting the availability and performance of the network [4]. The control plane of SDN has a lesser ability of scaling and allowing numerous request causing bottle necks on the network [5]. Although SDN has a denial-by-default security property to disallow and reject unauthorized users but a strong encryption key needs to be predefined to protect the actual data sent across the network [6]. Network load balancing is the implemented networking method deployed to distribute several client requests to resources through multiple servers. An efficient network performance was achieved [7] through a high-density SDN using load balancing. In [8] there was an efficient switch migration between multiple controllers of SDN deploying network load balance. For scalability, availability, and outmost performance (QoS) of an SDN, there is the need to deploy a Network Load Balancing technique (NLB) [9]. In this regard, we propose a new NLB technique which is a merger of a Hash IP load balancing algorithm with a Weighted scheduler (WS) and Dynamic switching of routing path (DSP).

The remainder of this paper is structured as follows: Section 2 details the literature review of load balancing in SDN. Section 3 explains the efficiency of each technique proposed in the HDW algorithm to be measured and tested. Section 4 of this paper shows the performance evaluation and comparison between other works after implementation of HDW algorithm. Section 5 draws the conclusions and recommendations for future work.

## Related works

SDN has been an area of growing interest in networking, with the potential of replacing legacy networks. There is much pressure and concern from industries to solve the basic challenges encountered by Software Define Network. There are many amazing surveys in SDN with Load Balancing, most of these surveys are carried out based on different perspectives [10]. For instance, M. R. Belgaum et al. [11] reviewed more than 20 pieces of literature on Load Balancing in SDN specifically in the sector of data centers, Gives no view of LB in SDN with a real or virtual network setup. A systematic approach for analyzing the algorithms and techniques of Load Balancing utilized by researchers were classified into traditional and artificial intelligence LB methods to solve the challenges in SDN. The various methodologies proposed could not standardize or resolve the challenges of SDN like throughput, overheads, congestion, etc. Hamed et al. [12] proposed a load balancing algorithm responsible for directing requests from clients to several servers. The LB algorithm enables the immediate request to be allocated to the least-loaded server on the network efficiently. The proposed least-loaded algorithm was evaluated by comparing the round-robin algorithm and the random algorithm. This algorithm proved to be effective as the throughput of the network improved, there was also a reduction in delays over the network. This paper did not consider the quality of services (QoS) metrics like congestions, response times, and bandwidth allocation. Robin Losch et al. [13] implemented a load balance strategy in a Distributed Hash Table to achieve a fair queue and optimal distribution resources. The actual size of packets (weight) to be allocated and execute was a great challenge. Weighted load balance with virtual and binary node algorithms was proposed to improve load scheduling, reduction of overheads and congestion. Due to multiple hashing, there are complexities in the algorithm and this affects the throughput and the performance of the network. Kaur et al. [14] proposed a Round-robin load balancing algorithm, which works with the first come first served approach. This algorithm provides a Uniform load distribution technique for every node on the network. The uniform distribution creates Static networks with rigid routing rules. Again there is massive delays over the network as a result of the non-dynamic nature of the Round Rubin algorithm. This affects the general performance, scalability and throughput of the network. Ramhani et al. [15] also introduced an efficient load balancing technique for routing on the network. Implementation of Multipath routing algorithm was deployed to create many routing paths for rerouting of loads. MRLB algorithm is very cost efficient. There is an effective resource allocation with better path decisions. There was no packet loss as a result of continuous rerouting of multiple loads on the network. This algorithm proves to be successful but there are a lot overheads and congestion which affects the performance of the network. Vyakaranal et al. [16] used a Weighted round-robin load balancing algorithm for their research work. The WRR algorithm gave an improved load scheduling mechanism. There was an efficient response time with maximum throughput. There is no starvation as every request is allocated to right server when More over this algorithm comes with complexities in re-routing. Wang et al. [17] developed a cross-domain load balancing mechanism for software defined networks in cloud data center. CDLB utilizes a dynamic technique for routing that improves the communication synchronization nodes on the network. Much emphasis was placed on how to manage the security and threats issues encountered on the network. Yilmaz et al. [18] proposed server load balancing technique to be used in software defined network for video streaming. Although this algorithm reduces delays and congestion, there are a lot of overheads on server with multiple loss of requests. The issues of overheads, delays, bottle necks and congestion of network requests and resources were challenges that was continuously encountered on the network by various researchers hence the deployment of the new Hash IP load balancing algorithm merged with the Weighted scheduler technique and Dynamic switching of routing path (HDW). The weighted scheduler allocates appropriate load of request to the right server to avoid overhead and to reduce delays and congestion on the network. Dynamic switching path functionality of the proposed HDW algorithm dynamically reroutes request to improve scalability to avoid bottle necks on the network. In the end, the proposed technique in this paper overcomes Hash Collisions and redirection of load limitations encountered in old Hash IP algorithms.

## The efficient NLB technique

Here, we develop the efficient NLB technique by merging the improved Hash IP algorithm with a Weighted scheduler and Dynamic switching of routing path (HDW algorithm). To improve security and network reliability, hash algorithm implements cryptographic function to hash data into unique key [19]. This helps to allocate resources to the right sever to prevent redirection and redundancy on the network [20].

### The improved hash IP algorithm

**Fig 1:**
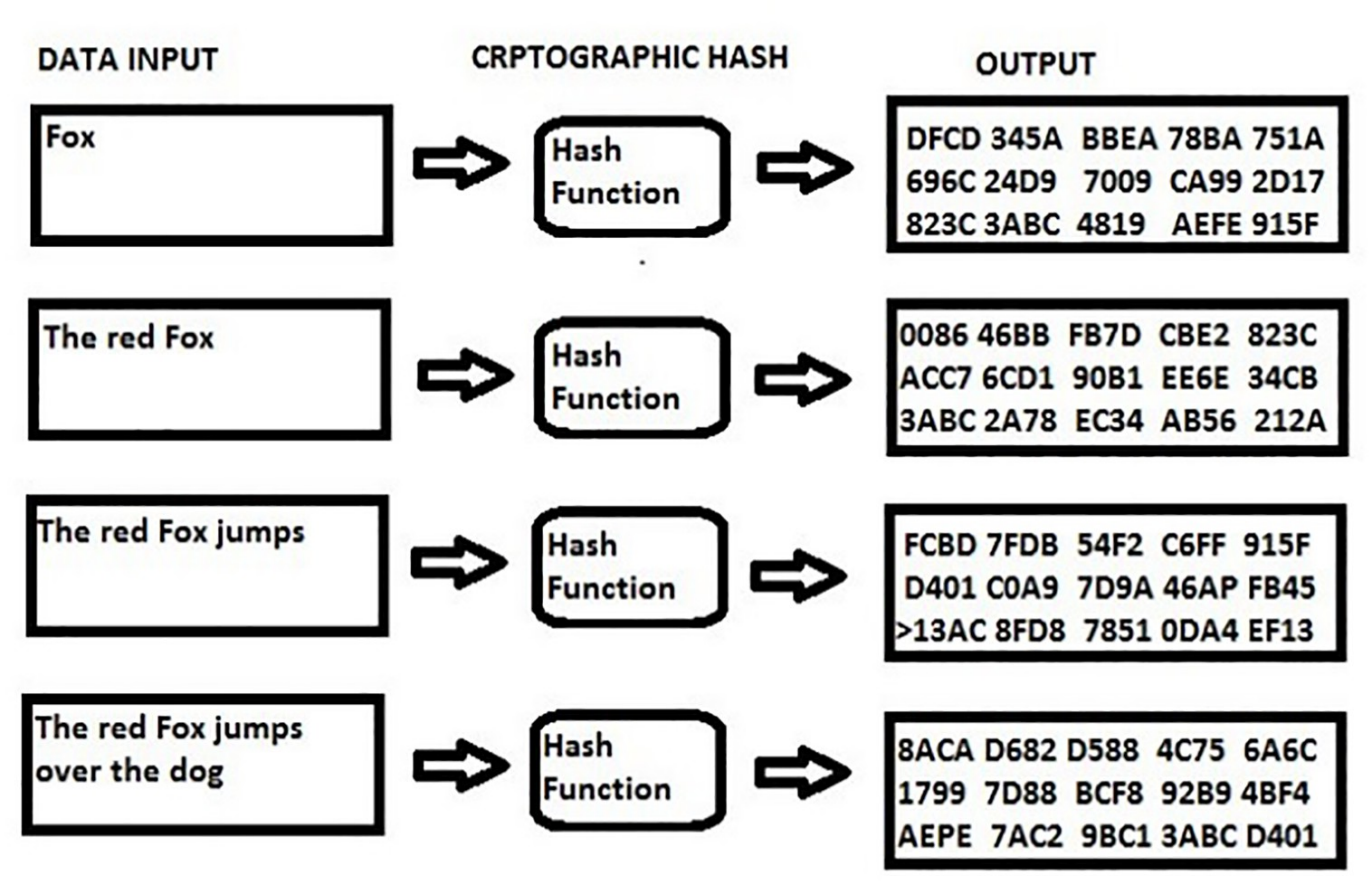
Cryptographic hash function.

The new Hash IP algorithm avoids the large table lookups of flows in Dynamic and Static algorithms. This network load balancing algorithm generates a unique hash key by combining the destination IP address and the source IP address of both client and server [21]. Unlike previous Hash IP algorithms, the new Hash IP algorithm adds the calculated weight of packets to form unique hash key to avoid Hash collisions which were major setbacks for previous Hash IP algorithms. The new formed hash key is used to allocate the client to a server. For variable range of bit length, secure hash algorithm is deployed by the network load balance for hashing. The cryptographic functions implement modular additions, compression formulas, and bitwise operations in the computation to transfer the data into hash values as shown in Fig 1. Incoming host IP addresses comes with IP header fields which provide basic information like the source IP, protocols, data, packet size, checksum etc. [22]. The size of packets is captured by the new Hash IP algorithm to calculate for the total weight of packets for the number of request queues on the network. The scheduling technique is adopted by Hash IP algorithm to enable appropriate request allocation on the network based on weight of packets.

### Weighted scheduling technique

This technique helps to disseminate traffics to the right servers on the network based on their strength. The actual size of packets (weight) to be transferred on the network was very difficult to determine by most weight scheduling techniques [23]. This limitation has been overcome by our proposed new hash IP algorithm. The new hash IP algorithm calculates for the total weight of packets to help the weight scheduler technique to allocate the right weight to the right servers on the network. These two methods work to complement each other. The weight scheduling technique first directs traffic with huge weight to the strongest server followed by the medium sever and to least one. With every queue *q*_*i*_ of an equal or different data packet size, there is an associate weight *w*_*i*_. Katevenis, Sidiropoulos, and Courcoubetis proposed the weight scheduling (WS) technique [24] which is represented by;

$$\begin{array}{c} \frac{w_{i}}{\sum_{k=1}^{n}w_{k}} \end{array}$$ (1)

For allocation of bandwidth, there is a consideration of the total weight and packet size of every incoming request. Having a mean *m*_*i*_ of each queue *q*_*i*_, the bandwidth allocation is calculated by;

$$\begin{array}{c} \frac{m_{i}\times w_{i}}{\sum_{k=1}^{n}m_{k}\times w_{k}} \end{array}$$ (2)

For link allocation of every queue *q*_*i*_, their capacity is

$$\begin{array}{c} \sum_{k=1}^{n}l_{i}=1 \end{array}$$ (3)

Therefore, weight is set as

$$\begin{array}{c} w_{i}=\frac{l_{i}}{m_{i}} \end{array}$$ (4)

Description of symbols for the equations is found in Table 1. For every weight, there is a calculated bandwidth and a specified link path to transmit the particular request on the network to avoid collision and to improve the network performance. In Hash tables, we deployed the insert, search and remove methods for hashing. The implemented Pseudocode methods created a unique way to control the values in the algorithm [25]. The values are the calculated weight of packets, data and the Key form by the IP addresses. The number of incoming requests (RN) channel in queues on the network are also added to the hash values to create hash difference and uniqueness.

**Table 1:** Notation table.

Symbol	Description
*w*_*i*_	Weight number of every packet on the network
*m*_*i*_	Mean of calculated weight
$\sum_{k=1}^{n}$	Sum of every weight
*l*_*i*_	Link path to direct packets
*n*, *k*, *i*	Variable to represent numbers

**Algorithm 1** New Hash Algorithm in Hash Table

1: Set Hash TB ← Hash Table

2: Set Data ← Data Value

3: Set Key ← Vector of IP addresses

4: Set Weight ← calculated weight of packets

5: Set RN(i) ← Number of incoming requests

6: Set h ← Hash Value {RN(i), Key, Data, Weight}

7: i = 0

8: Inserting values into Hash table

9: **if** Hash TB[h]== null or deleted **then**

10:  Hash TB[h]= new hash {RN(i), Key, Data, Weight}

11:  return h

12: **else**

13:  increment *i* = *i* + 1

14: **end if**

15: Searching and deleting values in the Hash table

16: g ← Hash.get {RN(i), Key, Data, Weight}

17: **if** g == null **then**

18:  return null

19: **else**

20:  when Hash TB[g]= Delete

21:  return g

22: **end if**

### Dynamic switching of routing path

We also proposed the best way of forwarding packets in the routing path of the software defined network. Implementation of conventional switches and programmed switches on the same network calls the efficient way to forward packets without leakages and breakages on the network [26]. Dynamic switching of a path (DSP) is the technique deployed which is resilient in nature for routing failures, conserves energy, and also extends the network’s lifetime [27]. DSP implements Hybrid routing protocols. The Hybrid routing protocol overcomes the difficulties faced in both static and dynamic protocols. The Hybrid protocols uses;

- Interior Gateway Routing Protocols (IGRP): Nodes with data were transmitted within the radius of the network. It utilized a maintained internal link state routing path and also implemented an enhanced IGRP for routing in autonomous systems.
- Exterior Gateway Routing Protocol (EGRP): Nodes with data were also transmitted outside the radius of the zone of the network. Erick Osborn, 2020) [28].

**Fig 2:**
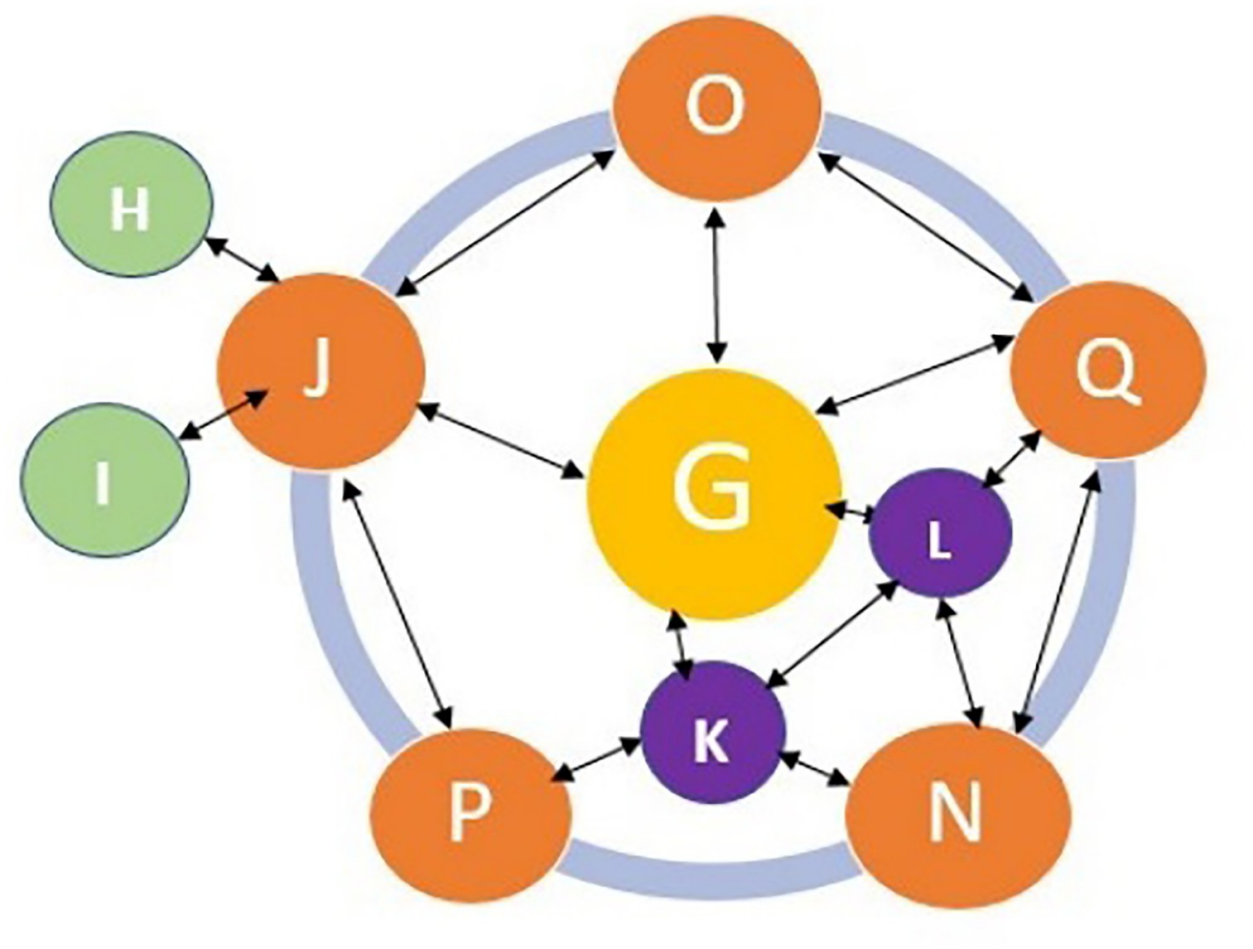
Zone routing of dynamic switching of the path.

Hybrid routing protocol with the dynamic protocols actively sets every node with zones and operates whenever there are transmissions inside or outside the network routing zones [29]. EGRP are for routing outer nodes and IGRP are for routing the inner nodes. In Fig 4, OQ is a Peripheral node which deploys an enhanced IGRP, K-L is interior nodes which also deploys IGRP. H and I are outer zones of G; node G transmits data outside the radius of the network by implementing the routing protocol using Exterior gateway routing protocol. Node G used border gateway protocol in EGRP to route outside the radius of network when data cannot be found within or inside the zone of the network. Fig 2 shows the routing mechanisms of DSP.

### Architectural module of HDW algorithm

**Fig 3:**
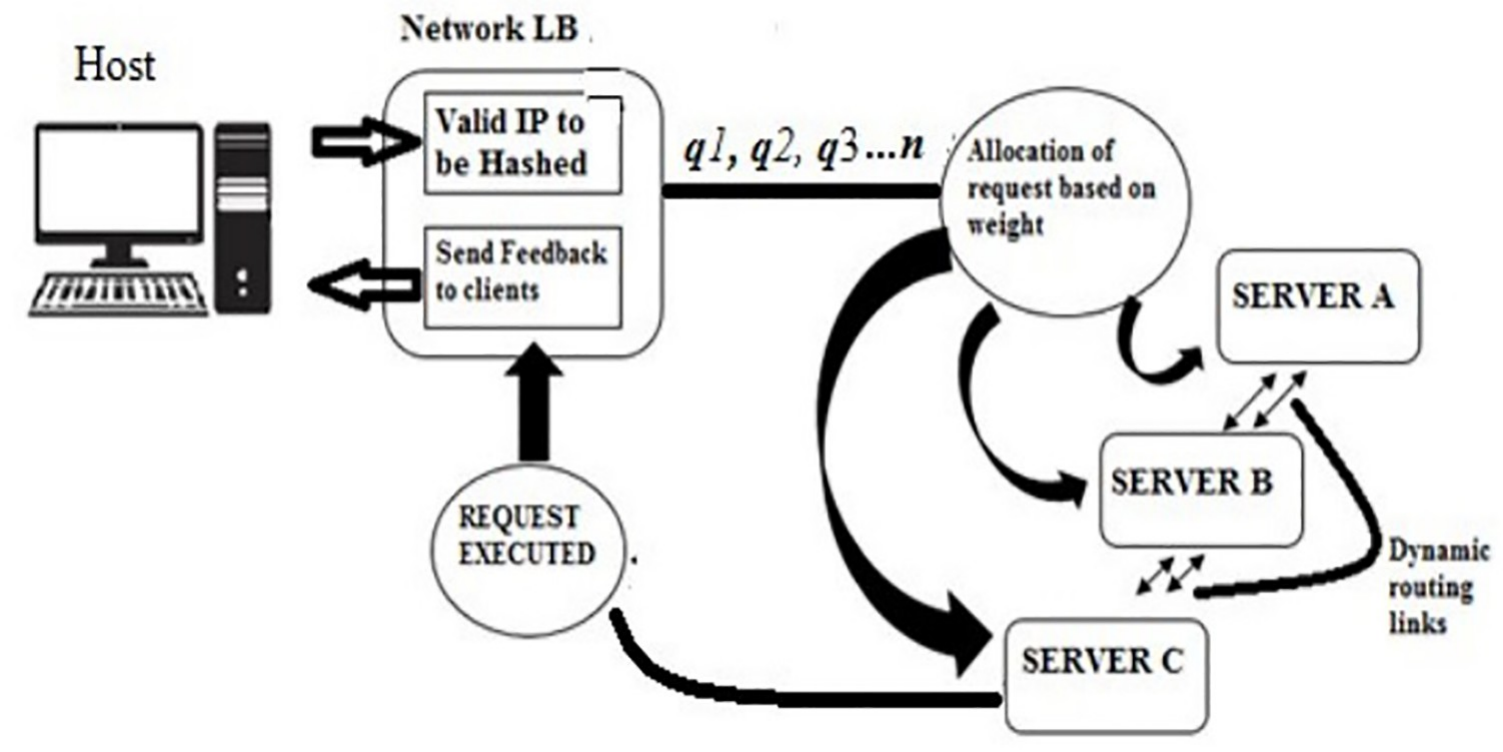
Architectural module of HDW.

An Architectural model of the HDW Algorithm displays the inter relationship between the merged techniques and how they operate to compliment and accommodate of each other on the network. In Fig 3, the number of requests is queued *q*_1_, *q*_2_, *q*_3_…*q*_*n*_ to the allocated server and every queue has an associated weight *w*_*i*_ (number of packets). If having three 3 servers on the SDN, server C will receive the higher loads of traffic which is followed by server B with medium loads of traffic and the least loads will be allocated to server A respectively. The dynamic links helps to dynamically switch routing path between servers to increase the efficiency of the network [30]. Floodlight controller provides the qualities to offload traffics on network as compared to other OpenFlow controllers which is evaluated during simulation.

## Performance evaluation

The network is evaluated based on the Quality of Service (QoS) processes like availability, scalability and the total performance of network with IPERF (internet performance) and Wireshark networking tools on metrics of throughput, response time, bandwidth and Jitters. We propose a tree topology which consists of 2 servers, 3 core switches, 4 aggregated switches, and 8 hosts. Host one (h1) pings/sends 20 packets to each of the seven hosts to measure the highest data and time taken to complete tasks in both first and second scenarios. The first scenario was tested without the network load balance on an Ubuntu virtual machine, the Second scenario was also tested with a floodlight controller as the remote controller on the Floodlight virtual machine. Bandwidth for iperf with floodlight controller ranged higher with 9.56 Mbits/sec to 10.7 Mbits/sec as compared to the ubuntu iperf without any controller which ranges from 8.07 Mbits/sec to 9.00 Mbits/sec. The iperf without a controller is relatively lower as compared to the iperf with a floodlight controller. The network without a controller gave priority to only one host at a time which delays and slows down the network causing congestion and jitters over the network. The floodlight controller approximately spread requests to several hosts on the network. It balances and directs all requests to the right servers over the network.

**Fig 4:**
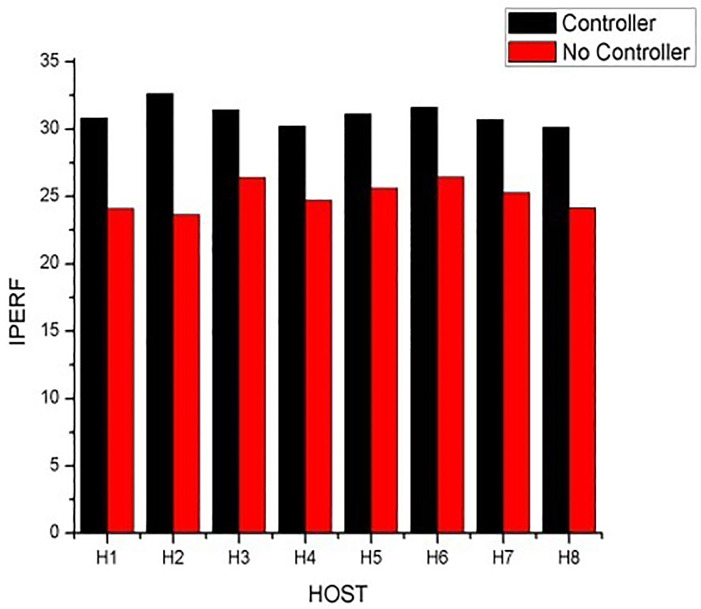
The average results of both networks using IPERF.

**Fig 5:**
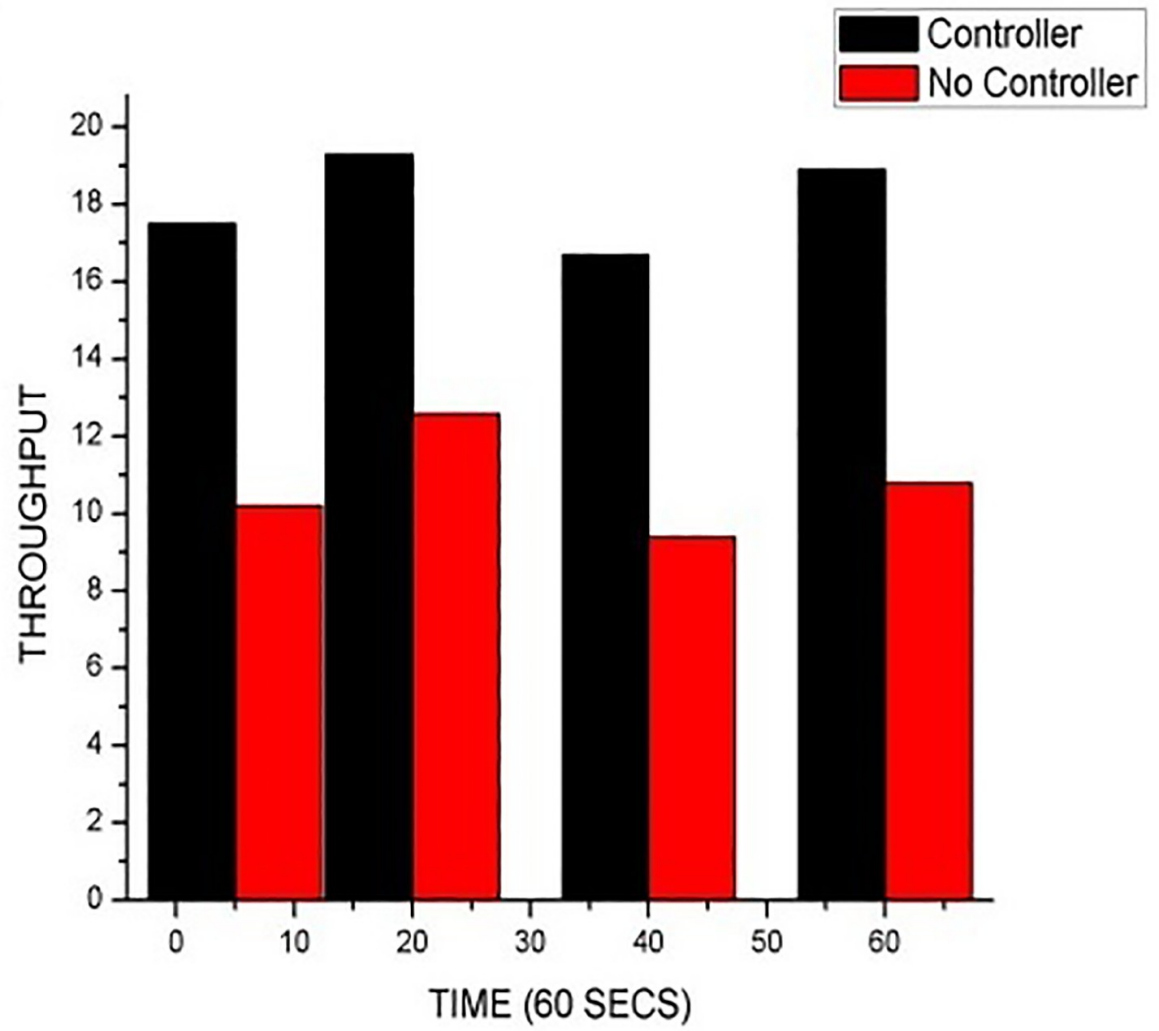
Throughput of 20 bytes transferred within 60 seconds.

Table 2 Shows both the internet performance (IPERF) of the network with the floodlight controller and also the one without any controller. After testing with IPERF, the network with Floodlight controller happens to obtain higher results with 5bits/sec more bandwidths as compared to the IPERF test of the network without a controller. Table 3 shows the average results after evaluating the QoS measures in Wireshark across the eight (8) hosts. Fig 4 represents the average results of both IPERF on a bar chart showing the difference between the network with a floodlight controller and the one without a controller. A graphical view is displayed. Fig 5 depicts the average throughput results after pinging 20 packets across the eight (8) hosts.

**Table 2:** Average Internet Performance (IPERF) of the network.

Host	IP Address	Average IPERF without Controller (Mbits/Sec)	Average IPERF with Controller (Mbits/Sec)
*H*_1_	10.0.0.1	24.11	30.80
*H*_2_	10.0.0.2	23.64	32.62
*H*_3_	10.0.0.3	26.40	31.41
*H*_4_	10.0.0.4	24.71	30.20
*H*_5_	10.0.0.5	25.62	31.11
*H*_6_	10.0.0.6	26.41	31.62
*H*_7_	10.0.0.7	25.28	30.70
*H*_8_	10.0.0.8	24.12	30.15

**Table 3:** The average results after evaluating the QOS measures in wireshark across the Eight (8) hosts.

Time (s)	No Controller			Floodlight Controller		
	Throughput (GB)	R. Time	Jitter	Throughput (GB)	R. TIME	Jitter
5	10.2	0.332	0.65	17.5	0.131	0.21
20	12.6	0.465	0.58	19.3	0.485	0.35
40	9.4	0.248	0.74	16.7	0.102	0.19
60	10.8	0.376	0.46	18.9	0.462	0.28

### Results comparison

In this section, we evaluate the performance of our proposed HDW algorithm by comparing it to three related works [3, 13, 16]. The related works refer to Vyakaranal et al.’s work [16] on distributed LB algorithm using WRR denoted as DWRR, Robin et al.’s work [13] on weighted load balance with binary node algorithm denoted as WLB and Mei-Ling et al.’s work [3] on dynamic weighted random selection algorithm denoted as DWRS respectively in Table 4. Moreover, we set equal parameters for each case of the experimentation with respect to each algorithm under consideration.

**Table 4:** Comparative analysis of related algorithms.

Research work	Performance Metrics				
	Throughput	Secured	Congestion Control	Availability	Jitter
DWRS [3]	✔	×	✔	✔	0.21
WLB [13]	×	✔	✔	✔	0.35
DWRR [16]	✔	×	×	✔	0.19
Proposed Technique	✔	✔	✔	✔	0.28

**Fig 6:**
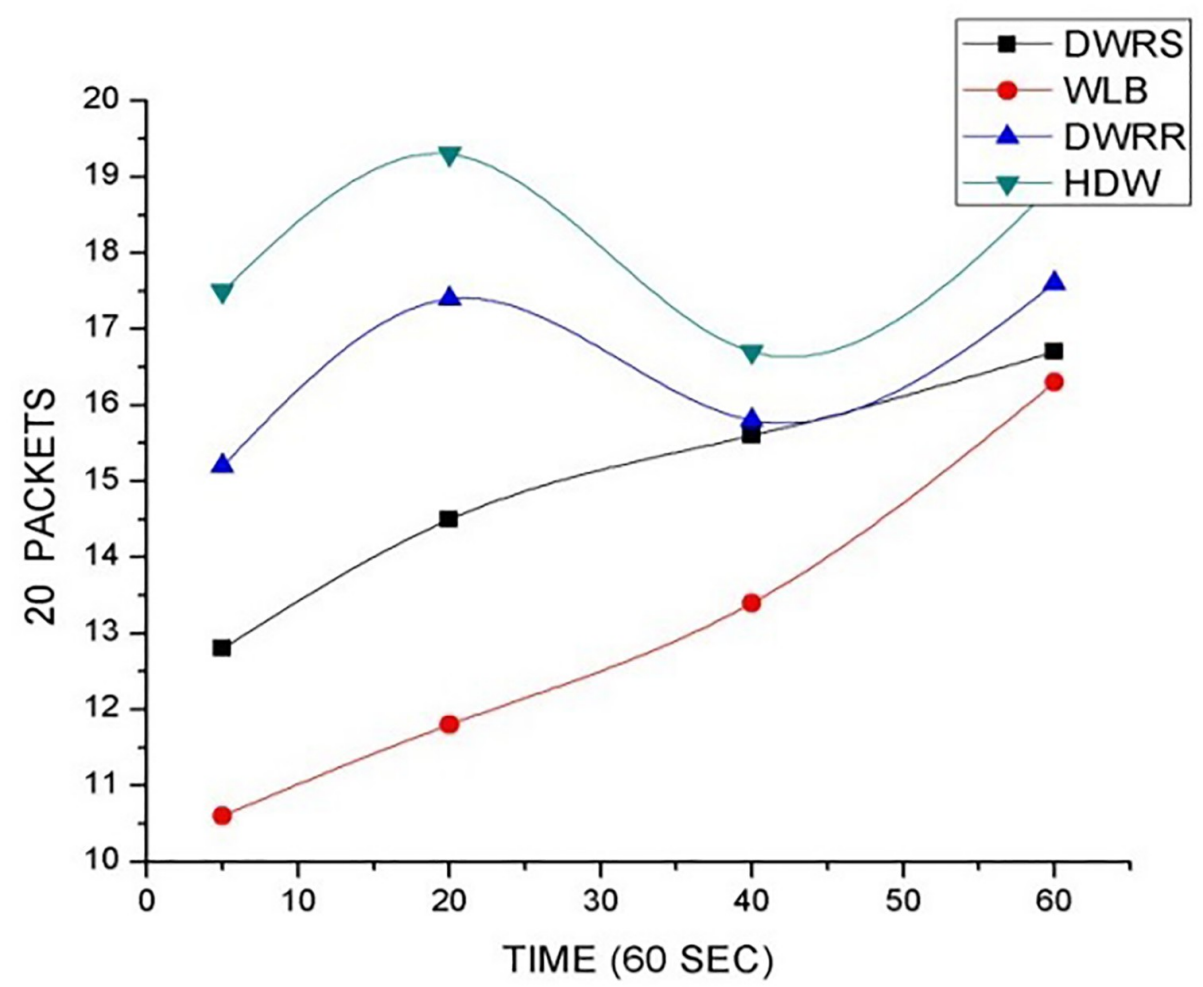
Comparative graph analysis of algorithms.

The performance metrics is based on quality of services like throughput, jitters, response time and security of the network. Our proposed HDW algorithm proved to be efficient as results obtained is relatively higher than the other compared algorithms. Vyakaranal et al.’s work [16] on distributed LB algorithm using WRR (DWRR), gave higher throughput with an improved network availability but it was not secured. Robin et al.’s work [13] on weighted load balance with binary node (WLB) algorithm was secured but it had lower resource availability which affected the network throughput. Mei-Ling et al.’s work [3] on dynamic weighted random selection (DWRS) algorithm randomly selects request to provide throughput and availability. Without taking consideration of weight and time of request execution, DWRS algorithm fairly affects congestion and it is not secured. Fig 6 shows the comparative graph analysis on proficient resource utilization of the various algorithms.

The proposed HDW algorithm provides maximum throughput to increase resource availability utilizing an efficient weight scheduling method. With the hash functionality, the HDW algorithm secures the network. Congestion is effectively controlled by implementing the dynamic switching of routing protocol.

## Conclusion

In this work, we deduced a network load balancing technique for SDN via a merger of a Hash IP load balancing algorithm with a weighted scheduler and dynamic switching of routing path. We found that in the simulation environment, the floodlight controller proved to be successful by showing the IP addresses for both source and destination, the scheduled links to direct traffics, and the total number of packets transmitted on the network. The proposed algorithm with floodlight controller gave higher results when throughput, response time and jitters were measured. It is a clear indication that utilizing dynamic network load balance with the new Hash IP algorithm merged with the weighted scheduling technique improves the scalability, availability, and performance of the Software defined network.

SDN has several controllers and in the near future, more research can be carried on the dynamic network load balance with other algorithms by implementing different SDN controllers to determine their performance outcomes. Other topologies with different sizes can be deployed on the network load balance with the new Hash IP algorithm and Weighted scheduling method to evaluate the limitations within the algorithm.
